# The Family Wellness Program: a bench to bedside translation of behavioral and social science research into a clinical program for intimate partners of warfighters following traumatic brain injury

**DOI:** 10.3389/frhs.2025.1575781

**Published:** 2025-08-13

**Authors:** Tracey A. Brickell, Megan M. Wright, Samantha M. Baschenis, Rael T. Lange, Jamie K. Sullivan, Louis M. French

**Affiliations:** ^1^ Traumatic Brain Injury Center of Excellence, Walter Reed National Military Medical Center, Bethesda, MD, United States; ^2^ National Intrepid Center of Excellence, Walter Reed National Military Medical Center, Bethesda, MD, United States; ^3^ Edward Hebert School of Medicine Uniformed Services University of the Health Sciences, Bethesda, MD, United States; ^4^ General Dynamics Information Technology, Falls Church, VA, United States; ^5^ CICONIX, Annapolis, MD, United States

**Keywords:** family, intimate partner, child, service member veteran, traumatic brain injury, neurobehavioral, health-related quality of life, treatment rehabilitation

## Abstract

This report details a bench to bedside translation of behavioral and social science research into a clinical program as a result of a collaboration between two United States Defense Health Agency Centers of Excellence for warfighter traumatic brain injury (TBI) and brain health. Identifying a gap in health-related quality of life (HRQOL) measures, our team instigated a 7-year multisite effort to validate and develop generic and caregiver specific HRQOL domains for family members of warfighters and civilians with a TBI using state-of-the-science measurement development standards; the *Traumatic Brain Injury Caregiver Quality of Life (TBI-CareQOL)* measurement system. The TBI-CareQOL was integrated into the Defense and Veterans Brain Injury Center-Traumatic Brain Injury Center of Excellence *15-Year Longitudinal TBI Study* designed to address four elements in a Congressional mandate (NDAA FY2007 Sec721 Public Law 109-364). Based on findings from the 15-Year Longitudinal TBI study and larger body of related literature demonstrating the bidirectional associations between warfighter neurobehavioral outcomes and family distress, relevant TBI-CareQOL measures were integrated into the *Family Wellness Program (FWP)* for intimate partner (IP) beneficiaries of warfighters with TBI in treatment for chronic neurobehavioral symptoms across the Defense Intrepid Network for Traumatic Brain Injury and Brain Health (DIN). The FWP screens IPs for clinically elevated HRQOL symptoms with clinical follow up offered in alignment with operations at each DIN treatment center and military base. In July 2024, the FWP was launched at the National Intrepid Center of Excellence at Walter Reed National Military Medical Center, and is currently expanding across the DIN.

## Introduction

Traumatic brain injury (TBI) was common in United States warfighters during the post-9/11 conflicts in Iraq and Afghanistan. Over 80% of TBIs sustained by warfighters since 2000 have been classified as mild in severity, though long-term complications are more likely following a more severe TBI (1). In the military, training accidents and combat experiences during the TBI event, and pre-and post-TBI, often contribute to the development of comorbid conditions that can result in significant disruption in physical, psychological, and social functioning. The symptom profile of co-occurring clinical conditions in warfighters can overlap with neurobehavioral symptoms following a TBI making it challenging to disentangle the etiology of persistent neurobehavioral symptoms, particularly following a remote mild TBI (MTBI). Some warfighters report neurobehavioral symptoms long after a TBI of any severity, and fail to reach desirable fitness for duty and quality of life outcomes (2, 3). Poor neurobehavioral outcomes increase the probability that a warfighter may not return to the operational environment and may require medical separation, or may return with reduced cognitive-behavioral capabilities and degrade unit readiness. Poor neurobehavioral outcomes may also lead to increased distress and dysfunction in the warfighter's home environment, particularly if coinciding with reintegration following a lengthy period of deployment separation (4, 5).

Warfighters with chronic neurobehavioral symptoms often require ongoing care, support, and advocacy from family members, most commonly their intimate partners (6–8). Chronic neurobehavioral symptoms have been consistently associated with poor health-related quality of life (HRQOL) and family relationships in military family members, regardless of TBI severity (9–13).

In 2006, the United States Congress passed H.R. 5122, also known as the John Warner National Defense Authorization Act (NDAA) for Fiscal Year (FY) 2007 (Public Law 109-364). Section 721 (Sec721) required “a longitudinal study on the effects of traumatic brain injury incurred by members of the Armed Forces serving in Operation Iraqi Freedom or Operation Enduring Freedom on the members who incur such an injury and their families”. Congress directed a study duration of 15 years, with reports on the results submitted to Congress on the 3rd, 7th, 11th, and 15th years (see health.mil/TBICoE15YearStudies). The Act specified four required elements to address; in short, an examination of [1] the long-term physical and mental health effects of TBIs, [2] the health care, mental health care, and rehabilitation needs of warfighters with TBI, [3] the type and availability of rehabilitation programs and services in the United States Departments of Defense and Veterans Affairs (DoD/VA), and broader community, and [4] the effect on family members.

**Figure 1:**
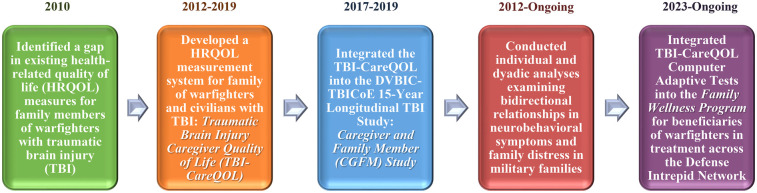
Process flowchart of translational behavioral and social science research into a clinical program. Acronyms: DVBIC-TBICoE = Defense and Veterans Brain Injury Center-Traumatic Brain Injury Center of Excellence. Defense Intrepid Network = Defense Intrepid Network for Traumatic Brain Injury and Brain Health (DIN).

In 2010, the Defense and Veterans Brain Injury Center-Traumatic Brain Injury Center of Excellence *15-Year Longitudinal TBI Study* was established to respond to NDAA FY2007 Sec721. Our team was assigned responsibility for the scientific development and oversight of the 15-Year Longitudinal TBI Study. As displayed in Figure 1, in the process of addressing NDAA FY2007 Sec721, our team [1] identified a gap in existing HRQOL measures for family members of warfighters with TBI; [2] developed and validated item banks to measure HRQOL in family members of warfighters and civilians with TBI using state-of-the-science instrument development standards; [3] integrated the measurement system into the 15-Year Longitudinal TBI Study; [4] conducted individual and dyadic analyses to identify measures most relevant for intimate partners (IPs) beneficiaries of warfighters with TBI and chronic neurobehavioral symptoms; and [5] based on findings from the 15-Year Longitudinal TBI study and larger body of literature, integrated relevant measures into a clinical program for IP beneficiaries of warfighters in TBI treatment across the Defense Intrepid Network for Traumatic Brain Injury and Brain Health (Defense Intrepid Network, DIN).

This bench to bedside basic science discovery translated into meaningful clinical practice was the result of an interdisciplinary collaborative effort between two United States Defense Health Agency networks within the Military Health System Centers of Excellence; namely [1] the Traumatic Brain Injury Center of Excellence (TBICoE), and [2] the National Intrepid Center of Excellence (NICoE) and larger DIN. This successful, productive, and collegial effort resulted in an empirically driven and innovative care initiative for DoD beneficiaries that has the potential for a direct effect on the health, wellbeing, and readiness of warfighters and their families.

The current report provides an overview of this 15 year effort and related literature including [1] the 15-Year Longitudinal TBI Study, [2] significant advances in patient reported outcome measurement and development of the TBI-CareQOL measurement system, [3] findings from the 15-Year Longitudinal TBI study and research from the larger body of related literature on health and wellbeing outcomes in TBI military caregiving families, and [4] the development and implementation of the FWP.

### The 15-Year Longitudinal TBI Study

The 15-Year Longitudinal TBI Study consists of two independent multisite longitudinal studies designed to address the four elements in NDAA FY2007 Sec721 including [1] the *Natural History of TBI Study (NH Study)*, and [2] the *Caregiver and Family Member Study (CGFM Study)*. Study evaluations started in 2010 and ceased in 2024. Both studies were housed at the NICoE at Walter Reed National Military Medical Center (WRNMMC) in Bethesda, Maryland, USA. Throughout the duration of the study, TBICoE sites across the research network were leveraged to support additional participant recruitment and evaluation efforts, including Naval Medical Center San Diego (California), Marine Corps Base Camp Pendleton (California), Joint Base San Antonio (Texas), Fort Bragg (North Carolina), and Fort Belvoir (Virginia).

The NH Study enrolled service members and veterans (SMVs) within three cohorts: [1] TBI (all severities), *n* = 1,061; [2] injured trauma controls, *n* = 493 and [3] non-injured controls, *n* = 221. Data were collected across six modules including blood, neuroimaging, neurocognitive, neurobehavioral, clinical interview, and sensory/motor. Data collection involved a combination of a two day in-person evaluation at the NICoE and a 1-to-2-hour remote telephone/web-based evaluation every 1–3 years. It is beyond the scope of this report to provide a comprehensive overview of this very large-scale study. However, a quick search for work by Dr. Rael T. Lange, the Scientific Director, in the published literature over the past 15 years should identify the many scientific outputs produced by the NH Study.

The CGFM Study is the main focus of the current overview. While a large body of work by the CGFM Study is covered in this report, it is also beyond the scope to provide a complete overview of this very large-scale study. A quick search of the literature over the past 15 years for work by Dr. Tracey A. Brickell, the Scientific Director, should identify additional scientific outputs produced by the CGFM Study.

In brief, the CGFM study enrolled family members who identified as providing care and support to a SMV with a TBI (all severities) diagnosed at a DoD/VA treatment facility, *n* = 587. Family members were also enrolled into two control cohorts including [1] family members who identified as caring for a SMV with posttraumatic stress disorder (PTSD) diagnosed at a DoD/VA treatment facility, *n* = 35, and [2] family members who did not identify as caring for the SMV, *n* = 44. The CGFM Study also invited SMVs from all cohorts to participate in the study with their IPs as a dyad sample, *n* = 244 dyads.

Recruitment was open to family or friends who self-identified as providing informal care for activities of daily living (ADLs; bathing, dressing, toileting), instrumental activities of daily living (IADLs; taking medication, running errands, balancing finances), and also non-ADLs/IADLs (emotional problems, aggression, pain management, legal advocacy, health care navigation). Professional caregivers with formal training and education in medical and non-medical care provision, or hired to provide medical care to the SMV were not included. The vast majority of participants were IPs of the SMV (90.8%), followed by parents (8.4%). Participants were recruited from extensive nationwide publicizing and networking, such as posting on social media, displaying flyers on information boards, and attending events and gatherings.

Participation involved annual completion of questionnaires through telephone/web-based procedures from a remote location during a scheduled appointment with a study investigator on the telephone to address any administration issues and quality control procedures. Completion of the questionnaires was self-directed by the participant, and for dyads (IP and SMV couples), occurred during the same appointment, but independent of each other.

### Development of a HRQOL measurement system for military family caregivers

Prior to starting the CGFM Study in 2010, significant advances had taken place in HRQOL measurement as part of a key initiative within the National Institutes of Health Roadmap process and Common Fund. These advances used state-of-the-science methods to create comprehensive measurement systems that addressed both [1] generic HRQOL, such as the *Patient Reported Outcomes Measurement Information System (PROMIS)*; and [2] HRQOL issues specific to various disorders, such as the *Quality of Life in Neurological Disorders (Neuro-QOL)* and *Traumatic Brain Injury Quality of Life* (TBI-QOL) measurement systems (14, 15). HRQOL is a subjective multidimensional construct that reflects the perceived impact a medical condition has on physical, psychological, and social wellbeing (16). The CGFM Study team was in need of a measure sophisticated enough to detect subtle changes in health outcomes longitudinally in family members, and one that would last the duration of a 15 year study. However, none of these existing measurement systems were developed or validated with family members of individuals with chronic conditions.

Identifying this gap in patient reported outcome measurement, the CGFM Study team became part of a 7-year multisite effort, involving civilian and military researchers, to validate existing HRQOL domains and develop caregiver specific HRQOL domains for family caregivers of SMVs and civilians with a TBI; the *Traumatic Brain Injury Caregiver Quality of Life (TBI-CareQOL)* measurement system. The development of the TBI-CareQOL took place over several phases using: [1] the PROMIS Instrument Development and Validation Scientific Standards throughout the development process, [2] a mixed methods research approach with both qualitative and quantitative data, and [3] a participatory action research (PAR) approach where family caregivers of SMVs and civilians with TBI were an integral part of development and validation procedures. See http://www.healthmeasures.net for more detail on measure development and research methodology for these comprehensive measurement systems.

In the first phase of the TBI-CareQOL development, a comprehensive literature review was conducted followed by qualitative research through focus groups with family caregivers of SMVs (17) and civilians (18). A thematic analysis of the literature and focus group data was conducted to identify HRQOL domains most relevant to caregivers. These domains included 17 existing domains through the PROMIS, Neuro-QOL, National Institutes of Health Toolbox, and TBI-QOL systems (e.g., Anxiety, Depression, Social Isolation, Emotional Support, Resilience, Sleep-Related Impairment). Eight new caregiver specific HRQOL domains were also identified (e.g., Caregiver Strain, Vigilance, Feeling Trapped, Loss, Emotional Suppression, Military Health Care Frustration). See http://www.tbicareqol.com for the complete list of item banks in the TBI-CareQOL domain framework. The focus group transcripts were used to develop the initial candidate items for the new caregiver specific HRQOL domains. Candidate items underwent an initial item-review process by the research team for item content feedback (e.g., wording, item overlap, appropriateness for the domain, and domain content coverage). Newly developed items that were redundant, confusing, and poorly written were eliminated (19–21).

During the next phase, the newly developed items underwent a qualitative item review process via telephone interviews with at least five caregivers per newly developed item. Caregivers provided feedback on the language and clarity of items and the relevance of the content. Newly developed items also underwent a Lexical Framework for Reading literacy review and revision to ensure they were no higher than a 5th grade reading level. Newly developed items additionally underwent a translatability review and revision to ensure items were able to be translated into other languages in future. Items that were higher than a 5th grade reading level or contained idiomatic language were reworded or eliminated (19, 20).

Existing and newly developed items banks were field tested with over 500 family caregivers of SMVs and civilians with TBI per item bank; with the exception of one military specific item bank (i.e., Military Health Care Frustration, *N* = 317 caregivers of SMVs). The reliability and validity of the existing PROMIS items banks was established for use in caregivers using various psychometric approaches, such as internal consistency reliability, item response theory based reliability, three week test-retest reliability, floor and ceiling effects, convergent validity, discriminant validity, known groups validity, clinical impairment rates relative to the normative sample, and effect sizes (22–25).

Data from field testing for the newly developed caregiver specific item banks underwent a series of analyses using specialized scaling techniques and psychometric approaches. Item response theory was used on each item to establish item calibration data (i.e., item parameters) needed to program a computer adaptive test (CAT) for each item bank. CAT technology provides an efficient method of administration. After a standard first item is administered, subsequent items are individually selected based on the response to the previous item, to provide an estimate of level of functioning using only a minimal number of items. The measurement precision of a CAT very closely approximates the precision of the full item bank, but only a small subset of the items (e.g., 4–6) in each bank are administered reducing participant burden (15, 19). A fixed short form containing 3–6 items was additionally developed for each new caregiver specific item bank by the research team using calibration-related statistics from the item response theory modeling, in combination with clinical considerations on item content and range of coverage (19). See (26, 27) for a more detailed description of the item banking process, item response theory, CAT and short form methodology, and its advantages in rehabilitation outcomes measurement. The reliability and validity of the CATS and short forms, and construct validity of the TBI-CareQOL measurement system was established (19–21, 28).

All newly developed and existing items use a Likert scale rating (e.g., never, rarely, sometimes, usually, always). Scores on all measures are transformed to a T-metric (M = 50, SD = 10). T-scores provide an interpretation of how a family caregiver is functioning relative to the reference sample (e.g., general or caregiving populations), making the TBI-CareQOL useful for both research and clinical practice (19). The TBI-CareQOL measures are publicly available free of charge on the HealthMeasures website (http://www.healthmeasures.net) without licensing or royalty fees. However, the Assessment Center Application Programming Interface is required for CAT administration and has an annual license fee. Access to a DoD server with the license for CAT administration was not an option at the time for the CGFM Study. As such, the TBI-CareQOL short forms were integrated longitudinally into the CGFM Study once finalized.

### Health and wellbeing outcomes in TBI military caregiving families: findings from the 15-Year Longitudinal TBI Study and related research

#### Related literature

TBI is a common combat and non-combat injury in members of the United States Armed Forces. Over 80% are classified as mild severity, with long-term complications more likely following a more severe TBI (1). In the military, training exercises and combat experiences often lead to the co-occurrence of physical and psychological comorbid conditions. Comorbid conditions may be associated with the TBI event itself or present pre-or post-injury. The symptom profile of many co-occurring clinical conditions often overlap with neurobehavioral symptoms and can result in significant disruption in physical, psychological, and social functioning, and fitness for duty in warfighters following a TBI of any severity. Mental health comorbidity, particularly PTSD, has been largely associated with persistent or newly developed self-reported neurobehavioral symptoms in SMVs up to 10 years following a TBI of any severity (2, 3, 29). Concurrent PTSD and TBI are very common in the military. PTSD was found to be one of the most significant and influential factors related to neurobehavioral outcomes, even more than TBI severity itself (3, 29).

SMVs with chronic neurobehavioral symptoms often require ongoing care, support, and advocacy from family members, frequently their IPs (7, 8, 30). Caregiving responsibilities are varied and can consist of help with activities of ADLs, IADLs, and non-ADLs/IADLs (31). Military family caregivers do not generally have formal medical education or training for managing neurobehavioral and comorbid symptoms, often leaving them overwhelmed and unprepared (32), particularly in the management of psychological health problems (33). Caregivers of SMVs with TBI often report providing care numerous hours per week for a range of activities, as well as managing other family, work, and household commitments, leaving little personal time for their own self-care (31). Caregivers tend to prioritize the wellbeing of the SMV above their own health needs, such as not getting adequate sleep, eating a healthy diet, engaging in adequate exercise, or taking part in regular health check-ups. They often allow their own health problems to reach a critical level before seeking care for themselves and place their own HRQOL at risk (32).

Research has accumulated documenting the negative association between care provision for SMVs with TBI and caregiver physical, psychological, social, caregiving, and economic HRQOL, and unhealthy family relationships, regardless of TBI severity (7, 9, 10, 30, 33, 34). The SMV's neurobehavioral symptoms and comorbid conditions have been consistently associated with worse caregiver outcomes, but not TBI severity. IPs report changes in the dynamic of their couples relationship with the SMV, navigating both care provision and romantic roles. Higher levels of caregiving distress were associated with divorce considerations (35). Several HRQOL constructs have been associated with protective qualities and reduced risk for poor outcomes in caregivers, such as resilience, life satisfaction, positive affect and wellbeing, self-esteem, social support, family relationships, and existential wellbeing (8, 9, 30).

Children of injured SMVs often receive less attention, emotional involvement, and positive parenting from the IP parent due to the demands of caregiving for the SMV parent (5, 36). Family members providing care to SMVs reported that care provision was a barrier to spending quality time with their children, created tension in the household, and impacted their parenting (32). Children in military caregiving families reported that their psychological health issues often went unnoticed due to the needs of the SMV and demands of caregiving (37). Children also reported taking on caregiving responsibilities that other children their age likely do not, leaving limited personal time for education, recreation, and social activities. For children who live in a two-parent household, it is possible that having one well-functioning parent may be protective and help moderate the risk for negative child outcomes. But if both parents are distressed, the negative impact on their children could worsen (38). While the negative association between parental combat deployment and child wellbeing during the post-9/11 conflict era has been well documented (39), health outcomes in children in TBI military caregiving families is sparse. In one study, children of warfighters with a range of injuries (38.9% TBI) had decreased preventative health care visits, but increased health care visits for mental health, injury, maltreatment, and psychiatric medication during the post-injury period, relative to pre-injury. The increase in visits was more pronounced in children of parents with PTSD, with and without TBI (40).

To date, research focused on a wide variety of factors thought to influence warfighter brain health and military readiness has saturated the literature, such as mental health factors, blast exposure, genomics, proteomics, access to services, sleep, headaches, and pain (41–45), but not influences in their family environment. TBI and psychological health conditions are often referred to as *invisible injuries*, because their symptoms are more difficult to associate with a medical condition compared to injuries and symptoms that are readily observable (e.g., bandages, limb loss, scars, prosthetic, wheelchair). Invisible injuries can complicate the family's understanding of why the SMV is experiencing difficulty resuming daily activities and re-establishing emotional bonds (5). TBI and psychological health conditions have been associated with higher levels of conflict and dysfunction in military families. In behavioral health treatment seeking SMVs, a majority reported some type of family problem, such as feeling like a guest in their home, children being afraid of them, disagreements over roles and responsibilities, marital discord, and shouting, pushing, or shoving (46). SMVs with a diagnosis of depression or PTSD were five times more likely to have family problems compared to SMVs without depression or PTSD. Emotional withdrawal, avoidance, and anxious symptoms were strongly associated with family problems. In treatment seeking SMVs at an outpatient clinic for SMVs with PTSD, TBI, and other mental health conditions, scores for family functioning were on average in the unhealthy range (47, 48). Unhealthy family functioning was related to lower parenting competence and PTSD emotional numbing and avoidance symptoms. Parents of military connected children with higher levels of stress, depression, and anxiety, and lower parenting competence reported worse family functioning (49). Worse family functioning was reported by SMVs with a TBI compared to SMVs without TBI (50). Having an unclassified TBI severity in the medical records, a comorbid condition, and combat experiences were related to worse family functioning, but not a moderate/severe TBI vs. MTBI.

#### 15-Year Longitudinal TBI Study

Overall, the findings from the CGFM Study were consistent with the broader TBI military caregiving family research and growing body of literature highlighting the importance of family wellbeing in warfighter recovery and return to duty following a TBI. The CGFM Study team conducted a series of individual and dyadic analyses throughout the 15-year duration of the study and the main findings are summarized below under three overarching themes.

##### Theme 1: warfighter neurobehavioral symptoms following traumatic brain injury are strong risk factors for poor HRQOL in their family caregivers

Over a series of analyses the CGFM Study found that many family members providing care to SMVs with TBI reported poor HRQOL outcomes across physical, psychological, social, caregiving, and economic domains (51–55). Longitudinally, many caregivers reported persistently clinically elevated symptoms or the development of clinically elevated HRQOL symptoms over time (56, 57). Among IP caregivers, worse HRQOL was related to unhealthy family functioning (58) and dissatisfaction in their couples relationship with the SMV (59). Some HRQOL domains were associated with a reduced risk for negative HRQOL outcomes, such as higher levels of resilience, life satisfaction, and emotional support, and lower feelings of rejection (53, 60, 61).

Caregivers reported caring for a range of ADLs, IADLs, and non-ADLs/IADLs (52). Poor health and family outcomes were consistently associated with SMV neurobehavioral symptoms related to adjustment (e.g., anxiety, depression, aggression, pain, fatigue, relationships), but not ability (e.g., mobility, vision, speech, memory, attention/concentration) (12, 51, 53, 58–60, 62–64). The findings for TBI severity were less consistent (58, 60, 62, 63). In one study specifically examining differences between caregivers of SMVs with a MTBI compared to more severe TBI, worse HRQOL was found for caregivers of SMVs with a MTBI (12). Caregiving for persistent neurobehavioral symptoms was likely interrelated with the high prevalence of comorbid conditions (e.g., PTSD, depression, pain, headache, substance use, sleep disorders). SMV injury characteristics (e.g., mechanism, hospitalization, years post-injury/caregiving), SMV military characteristics (e.g., combat experiences, combat deployments, military branch, pay grade), and family sociodemographic characteristics (e.g., income, race, education, age) also received less consistent support with caregiver health and family outcomes (6, 12, 58, 60, 62, 63).

In a subsample of caregivers who self-identified as no longer providing care to the SMV, no longer being in an intimate relationship with the SMV was one of the most frequently cited reasons for no longer caregiving (53). Compared to those still caregiving, family members who were no longer caregiving reported lower satisfaction in their intimate and caregiving relationships 12 months prior to first identifying as no longer being a caregiver. Improvement in HRQOL was found within the first 12 months of no longer caregiving.

##### Theme 2: parental distress and family dysfunction are strong risk factors for poor HRQOL in children of warfighters with TBI

The CGFM Study also examined HRQOL in children living in TBI military caregiving families, with one parent the SMV with TBI and the second parent the IP and caregiver of the SMV. Using IP parent proxy pediatric report, a high prevalence of children in TBI military caregiving families had clinically elevated HRQOL symptoms (65). Over half of the children were living in a home with both parents experiencing high distress. The SMV's neurobehavioral symptoms were singularly associated with worse HRQOL in their children. High distress in the IP parent providing care to the SMV was associated with further impairment in the HRQOL of their children. There was a trend for worse pediatric HRQOL when both parents had high levels of distress. Family sociodemographic characteristics were not consistently associated with negative pediatric HRQOL outcomes (65).

Examining psychological health longitudinally, many children had persistently clinically elevated or developed clinically elevated psychological symptoms over time.^1^ Parental distress in the SMV and IP, and unhealthy family functioning were associated with both the development and persistence of clinically elevated psychological symptoms in their children longitudinally. There was also a strengthening effect for these family risk factors on pediatric psychological health over time. The largest effects were generally found for IP psychological, social, and caregiving HRQOL, followed by SMV neurobehavioral adjustment symptoms.

##### Theme 3: intimate partner and family distress are strong risk factors for poor warfighter brain health following traumatic brain injury

Until recently, the majority of military TBI caregiving research had focused on how the warfighter's neurobehavioral symptoms were associated with negative outcomes in their family members. Little attention had been given to how the family's affective, behavioral, and social dysregulation may be related to poor neurobehavioral outcomes in warfighters. The relationship between warfighter and family distress is likely to bidirectional, where neurobehavioral symptoms influence and are influenced by family distress. Recent individual and dyadic studies by both the CGFM Study and the NH Study demonstrated the strong relationship between IP and family distress with poor neurobehavioral outcomes.

Using data from the NH Study, our team demonstrated the very strong negative association of an unhealthy family environment with warfighter brain health, particularly when recovering from a TBI.^2^ We found that a SMV with a MTBI living in an unhealthy family environment was 9.8 times more likely to have poor neurobehavioral outcome compared to non-injured healthy controls living in an unhealthy family environment, and 28.1 times more likely to have poor outcome compared to healthy controls living in a healthy family environment. Similarly, a SMV with a more severe TBI living in an unhealthy family environment was 5.9 times more likely to have poor neurobehavioral outcome compared to healthy controls living in an unhealthy family environment, and 16.9 times more likely to have poor outcome compared healthy controls living in a healthy family environment.

Using longitudinal data from the CGFM Study, our team found that clinically elevated scores on measures of physical, psychological, social, and caregiving HRQOL in IPs were very strong risk factors for chronic neurobehavioral symptoms in their warfighters following a MTBI (66). The CGFM Study additionally found that IPs of warfighters who were receiving treatment at the NICoE intensive outpatient program reported a worsening longitudinal trend in clinically elevated symptoms on many of these IP HRQOL risk factors for chronic neurobehavioral symptoms (57). In a merged dyad sample of SMVs enrolled in the NH Study and their adult family members enrolled in the CGFM Study, we found that a range of family member reported physical, psychological, and social HRQOL, and family functioning risk factors were strongly associated with SMV reported clinically elevated chronic neurobehavioral symptoms following a TBI of any severity (13). The findings for IP HRQOL and family dysfunction as risk factors for chronic neurobehavioral symptoms were replicated in an independent dyad sample of SMVs with MTBI and their IPs from the CGFM Study (67). The CGFM Study dyad sample additionally included the SMV's report on family dysfunction, which was found to be a very strong risk factor for chronic neurobehavioral symptoms. For example, SMVs were [1] 13.0 times more likely to have poor neurobehavioral outcomes when they had negative family experiences vs. positive family experiences; [2] 10.6 time more likely to have poor neurobehavioral outcomes when they had unhealthy family functioning vs. healthy family functioning; and [3] 4.2 times more likely to have poor neurobehavioral outcomes when they were dissatisfied vs. satisfied in their relationship.

Additional CGFM Study dyadic analyses examining couples satisfaction revealed that close to a third of both members of a dyad were dissatisfied in their intimate relationship (68). Couples dissatisfaction was associated with worse HRQOL, neurobehavioral, and family outcomes. SMVs tended to report worse outcomes compared to their IPs, except when the SMV was satisfied and the IP was dissatisfied. Dissatisfied SMVs reported worse outcomes compared to satisfied SMVs, and dissatisfied IPs reported worse outcomes compared to satisfied IPs.

##### Research from military couples with warfighter PTSD suggests that family member behavioral and emotional accommodations may undermine warfighter recovery and readiness

Individual and dyadic analyses by other research teams investigating family dynamics in military couples with warfighter PTSD had relevance to the interpretation of the CGFM Study and NH Study findings and the larger body of literature. Warfighters experiencing emotional numbing, withdrawal, and avoidance symptoms can be emotionally distant, and less likely to engage in intimacy and emotional communication. When these symptoms are internalized by their IPs, they can be attributed to a lack of love or demise in the couples relationship and lead to relationship and psychological distress (69). In an attempt to manage or reduce the warfighter's PTSD symptoms, IPs often accommodate their emotions and behaviors, such as avoiding contentious conversations, intimacy, social situations, and household noise, and assuming household chores, roles, and responsibilities previously shared with the warfighter (69, 70). While often well-intentioned, accommodative behavioral and emotional actions may inadvertently reinforce or facilitate the warfighter's symptoms, undermine recovery, and impede warfighter military readiness. Accommodative behavioral and emotional actions can also lead to elevated IP psychological, social, caregiving, relationship, and family distress (69, 70).

Some researchers have started including intimate partners in cognitive-behavioral rehabilitations programs using a couple-based or conjoint intervention design to address health and family issues in military couples as a dyadic approach to treatment outcomes. *Cognitive-Behavioral Conjoint Therapy*, and the online, self-guided version (*Couple Helping Overcome PTSD and Enhance Relationships*), is a couples therapy using dyadic cognitive-behavioral approaches to the treatment of warfighter PTSD (71–74). These conjoint cognitive-behavioral interventions have been helpful in reducing psychological and relationship distress, social and communication avoidance, and use of accommodations in military couples with warfighter PTSD. Dyadic cognitive-behavioral approaches may be helpful for improving HRQOL, return to duty, and readiness outcomes in military couples with SMV TBI.

PTSD and other mental health symptoms have overlapping symptoms profiles with neurobehavioral symptoms. Previous qualitative research revealed that IPs of warfighters with TBI often described engaging in similar behavioral and emotional accommodative actions (33, 36), including family members in the CGFM Study (17, 75). Family members in the CGFM Study focus groups frequently discussed suppressing their emotions; experiencing a loss of companionship, emotional support, and intimacy; and feeling rejected and isolated. Caregivers reported adopting behavioral and emotional actions centered around anticipating and managing problems before they escalated into emotional or physical reactions from the SMV. Caregivers reported feeling like they could not leave the SMV for an extended period of time, and experiencing heightened alertness from constantly monitoring the environment and controlling their behavior and the behavior of others in order to avoid emotional and physical aggression from the SMV toward family members and others. These behavioral and emotional actions resulted in feelings of vigilance and anxiety, and withdrawal from social, leisure, family, and employment activities. These accommodations were linked the high prevalence of co-occurring PTSD.

Overall, these family dynamics may have important implications for the treatment of acute and chronic neurobehavioral symptoms in warfighters with TBI and psychological health comorbidities. If a warfighter is discharged from treatment to a home environment with high levels of distress and dysfunction, improvement in symptoms and return to duty outcomes may diminish over time, because conflict, disorganization, and poor affective, behavioral, and social regulation within their family may undermine treatment outcomes. In response to this growing body of research demonstrating the bidirectional associations between chronic neurobehavioral symptoms and family distress, and success in dual-goal dyadic approaches to warfighter treatment, DIN leadership connected with the Scientific Director of the CGFM Study to integrate relevant TBI-CareQOL measures into the establishment of a *Family Wellness Program (FWP)* for IP beneficiaries of warfighters receiving treatment for chronic neurobehavioral symptoms.

### Development and implementation of the Family Wellness Program

The FWP was developed by CGFM Study leads, with NICoE clinician input, NICoE information technology and informatics support (IT), and feedback from TBICoE clinical researchers across the DIN. The NICoE had the Assessment Center Application Programming Interface license at time of the development, allowing for access to, and administration of the TBI-CareQOL CATS in the FWP. The FWP includes select item banks from physical, psychological, social, and caregiving HRQOL domains as outlined in Table 1. Item banks that had undergone Spanish translation and linguistic validation were also integrated into the FWP.

**Table 1:** Family Wellness Program health-related quality of life domain framework.

HRQOL Domain	
Physical HRQOL	Social HRQOL
Pain Interference^b^	Ability to Participate in Social Roles and Activities^a^^,^^b^
Sleep-Related Impairment^b^	Social Isolation
Fatigue^b^	Emotional Support^a^^,^^b^
Psychological HRQOL	Caregiver Specific HRQOL
Anxiety^b^	Caregiver Strain
Depression^b^	Caregiver Vigilance
Anger^b^	Emotional Suppression
Meaning and Purpose^a^	Feeling Trapped
	Feelings of Loss (Self and Person with TBI)
	Military Health Care Frustration

HRQOL, health-related quality of life.

^a^ Lower scores reflect worse functioning.

^b^ Spanish translation and linguistic validation.

The FWP is administered on the TBI Portal. The TBI Portal is a Defense Health Agency enterprise application within CarePoint; a health information delivery portal for the United States Military Health System. This Common Access Card enabled and password-protected application provides a consolidated view of TBI patient data that informs clinical care decisions and treatment planning across the Military Health System.

The FWP CATS can be completed by the IP in-person at the TBI clinic or remotely from the comfort of their own home by scanning a quick-response code via a smart phone or selecting a link sent via e-mail. It takes approximately 15 minutes to complete the measures, although administration time depends on the CAT stopping parameters and rules for each measure (see http://www.healthmeasures.net). The clinician receives an email once the IP has completed the measures.

**Figure 2:**
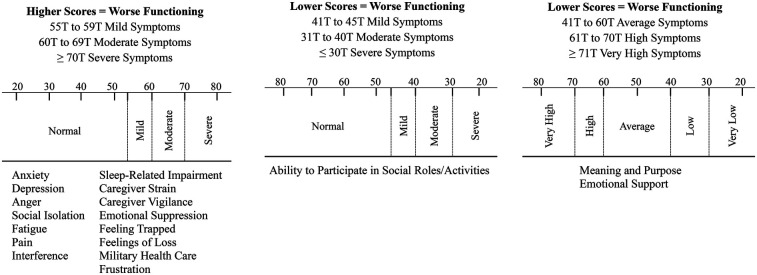
Healthmeasures cut-scores to interpret T-scores.

A Clinical Interpretation Manual was developed with information on reference populations for T-score generation for each item bank and cut-scores recommended by HealthMeasures to assist clinicians in interpreting T-score symptom severity (e.g., normal, mild, moderate, severe); see Figure 2. A quick look up guide containing the name of the item bank, example items to orient the clinician quickly to the content of each item bank, and cut-scores with severity ratings was also included in the manual. For illustration: ITEM BANK = Caregiver Strain; EXAMPLE ITEM = “I feel like there is no rest when it comes to providing care for the person with the injury;” CUT SCORES = ≤54 T Normal Symptoms, 55 T to 59 T Mild Symptoms, 60 T to 69 T Moderate Symptoms, ≥70 T Severe Symptoms. The cut-score severity ratings were additionally integrated into the TBI Portal clinician administration platform. The T-score symptom severity ratings are automatically generated in the TBI Portal after the IP completes each item bank, negating the use of the look up guide and reducing clinician burden. It is important to note that the cut-scores are not diagnostic and serve as a screening guide to identify elevated scores. Further diagnostic interviewing is required for any IP identified with elevated scores to reach a formal diagnosis.

**Figure 3:**
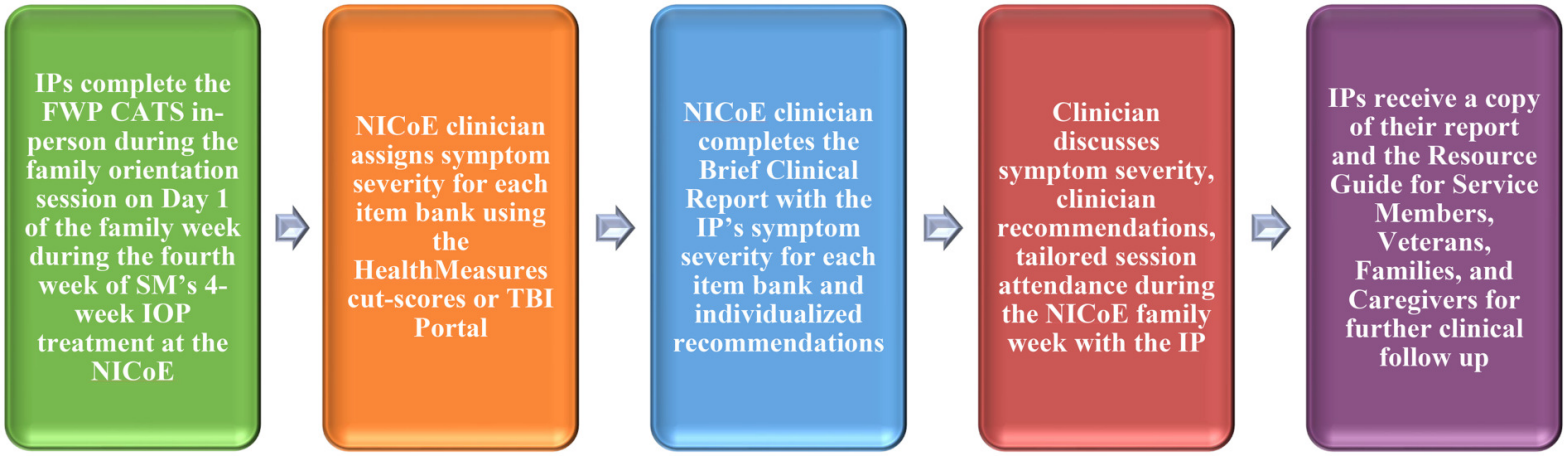
Process flowchart of the The Family Wellness Program at The National Intrepid Center of Excellence, Walter Reed National Military Medical Center. Acronyms: IP = Intimate Partner. CATS = Computer Adaptive Tests. IOP = Intensive Outpatient Program. NICoE = National Intrepid Center of Excellence.

The FWP was launched at the NICoE in July 2024 with IP beneficiaries of warfighters receiving treatment in the intensive outpatient program. The intensive outpatient program treats warfighters with TBI and psychological health symptoms who have plateaued in their recovery, with the goal of symptom improvement and return to full duty following treatment (76). The NICoE intensive outpatient program model of care includes a 4-week program that uses neurological and behavioral health treatments spanning 17 conventional rehabilitation and integrative medicine disciplines (e.g., neurology, neuropsychology, physical therapy, audiology, speech and language, optometry, nutrition, acupuncture, creative arts, mind-body, and animal assisted). Family members are invited during the fourth week and take part in the already established Family Program and primarily education-based sessions. After the IP completes the CATS, a brief clinical report is completed by the clinician in live-time and reviewed by the clinician with the IP. The report includes a clinical interpretation of the IP's symptom severity (normal, mild, moderate, severe) and clinical recommendations, such as potential treatment options on base and in the community, referral needs and pathways, and tailored session attendance during the family week at NICoE. IPs receive a copy of the report to take to their primary care provider for referrals to clinical services if needed. Intimate partners are also provided with resource directories containing information about national and local resources and programs for military families. Figure 3 displays the process flow for the FWP at the NICoE. Now operational, the NICoE has discussed extending the FWP to IPs of warfighters in other outpatient programs.

The FWP is also expanding across the larger DIN. The DIN is a program of record included in Section 721 of the National Defense Authorization Act for Fiscal Year 2025 (NDAA FY2025 Sec721). The DIN is a Defense Health Agency network of clinical care, research, and education that addresses the full spectrum of brain health through a patient centered, holistic interdisciplinary model of care. In addition to the NICoE, the DIN includes 10 Intrepid Spirit Centers (ISCs), and two TBI and Brain Health Clinics (see http://www.health.mil).

**Figure 4:**
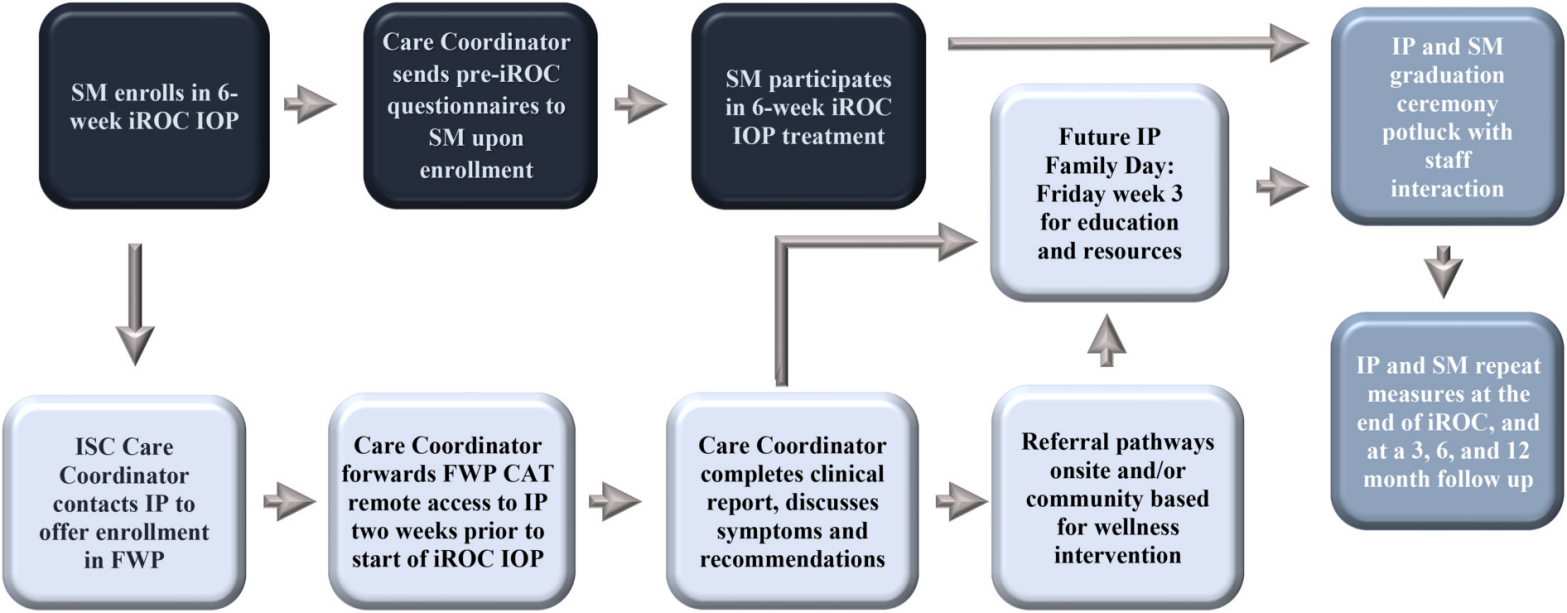
Dyadic process flowchart of the Family Wellness Program at the Fort Carson Intrepid Spirit Center, Evans Army Community Hospital. SM, service member; IP, intimate partner; CAT, computer adaptive test; IOP, intensive outpatient program; ISC, intrepid spirit center; FWP, family wellness program.

Each DIN site can access the FWP CATS through the TBI Portal. Site-specific clinician and IT input will guide administration and clinical follow up operations offered at each DIN treatment center. Expansion across the DIN started with the ISC at Fort Carson (Evans Army Community Hospital, Colorado). The Fort Carson ISC did not have any existing services for IPs prior to establishing the FWP. The FWP at Fort Carson will initially be offered to IPs of warfighters in the ISC 6-week intensive outpatient program, with potential expansion to other outpatient services in future. The IPs complete the FWP CATS remotely at the same time that the service member (SM) completes intensive outpatient program questionnaires, approximately two weeks prior to participation, at the end of the 6-week program, and at a 3, 6, and 12 months follow up. In the future when clinician resources allow, the Fort Carson FWP would like to establish a dedicated Family Day on the Friday of the third week of the intensive outpatient program. IPs will be invited to the ISC to participate in education sessions and receive handouts with local and national resources. In the meantime, IPs are invited to an already established event; their SM's graduation ceremony and potluck on the last day of the intensive outpatient program. During the event, IPs have time to interact and follow up with clinical staff. The FWP launched at Fort Carson ISC in January 2025. Figure 4 displays the dyadic process flow for the FWP and intensive outpatient program at the Fort Carson ISC.

Expansion efforts of the FWP across the DIN have also extended to the ISC at Camp Pendleton (Naval Hospital Camp Pendleton, California). Camp Pendleton ISC launched the FWP in January 2025 with a model similar to Fort Carson ISC. A clinical recommendation from recent CGFM Study and NH Study publications to include dyadic cognitive-behavioral interventions in the FWP for maintenance of treatment outcomes and military family readiness post-discharge (13, 66–68) (see text footnote 2) is being enacted upon by the Camp Pendleton FWP clinical provider. The clinical provider participated in Cognitive-Behavioral Conjoint Therapy training in anticipation of integrating couples therapy using dyadic cognitive-behavioral approaches into the Camp Pendleton FWP and eventually expanding across the DIN.

Fort Bragg ISC (Womack Army Medical Center, North Carolina) has established the infrastructure for the FWP and plans launch in September/October 2025. Joint Base Lewis-McChord ISC (Madigan Army Medical Center, Washington) will also move forward and establish the FWP onsite. The specific launch date is still to be determined but estimated for early 2026 (see Sustainability and Limitations below).

In addition to the TBI-CareQOL item banks, the TBI Portal provides access to the vast collection of English and Spanish adult, pediatric, and parent proxy pediatric generic and disorder specific HealthMeasures CATS for clinical and research application. Access includes CATS from the TBI-QOL and Neuro-QOL measurement systems developed and validated for individuals with TBI and other neurological conditions using the PROMIS Instrument Development and Validation Scientific Standards. Select PROMIS, TBI-QOL, and Neuro-QOL measures are being integrated into the SMs assessment battery across DIN sites. The accessibility to these comprehensive measurement systems and use of standardized scores (T-scores) allows for scores on measures to be directly interpretable across individuals. For example, researchers and clinicians using the measures can compare SM to SM, IP to IP, or SM to IP within and across DIN sites (26).

### Referral pathways for clinical services

A wide range of formats exist of interventions for improving the wellbeing of military family members; see (77–79) for a comprehensive review of interventions, theorical underpinnings, and empirical evidence on the effectiveness for individuals, couples, and families. Overall, positive benefits for caregiver HRQOL and family outcomes have been found for military family members who participated in interventions aimed at improving the caregiver's health and wellbeing, understanding of the SM's condition, care provision skills, problem solving, stress management (80, 81), mindfulness, relaxation (82, 83), couples relationship satisfaction (71–74), and family functioning (84–86).

To better support the needs of United States military caregivers, in 2010 the Caregivers and Veterans Omnibus Health Services Act (Public Law 111–163) legislation was enacted. In response, the VA established the most comprehensive family caregiver program ever enacted in the United States; the Caregiver Support Program (CSP) (87). The CSP is comprised of two programs. The Program of General Caregiver Support Services provides general services such as skills training, stress management, self-care, and respite care. The Program of Comprehensive Assistance for Family Caregivers offers additional services such as a monthly financial stipend, health care, and reimbursement for travel expenses for caregivers of seriously injured and ill veterans with a VA service-connected disability ≥ 70% (including TBI and psychological trauma) and require extensive in-person care. Positive impacts for caregiver HRQOL and veteran healthcare utilization have been reported by those enrolled (87, 88).

However, the training and other benefits and services offered through the CSP are only available to caregivers of veterans in VA health care. Yet, caregiving is not limited to veterans. Our team found that IPs of SMs with a TBI reported providing ongoing care, support, and advocacy for numerous years while the SM was still in the military (52, 57). Many IPs who were providing care and support to a SM reported clinically elevated scores across physical, psychological, social, and caregiving HRQOL domains. In addition, there was an increasing trend in the number of family members who developed clinically elevated symptoms over two years (57).

The DoD offers some resources to family members providing care, such as a resource directory, forums for caregiver peer networking, and caregiver support coordinators. However, the scope of services appears to be limited in comparison to the CSP. In previous research with IPs of SMs, various physical, psychological, and social HRQOL needs were reported, yet challenges navigating and accessing resources resulted in unmet needs (89, 90). Services tended to be *ad hoc* and site specific, and IPs experienced difficulty finding appropriate resources and accessing them.

At the time of writing this report, the FWP was operating primarily as a screening program. If an IP reported elevated scores on a measure, recommendations for clinical intervention were identified, discussed with the IP, and included in their clinical report. If self-referral was not an option, the IP could take the report to their primary care provider for a formal referral. In the absence of a centralized system of care like the VA CSP in the DoD, the FWP has established referral pathways for IPs to several programs that cater for the unique needs of military families within the DoD and community. The goal of the referral pathways is to have the IPs receive wellness intervention at the same time the SM is participating in the intensive outpatient program, aiming for a dyadic approach to TBI treatment and outcomes. For example, the DoD Armed Forces Wellness Centers offer health and wellness education, coaching, and fitness testing across a range healthy lifestyle programs to facilitate readiness and resiliency in military families, such as substance use, chronic conditions, nutrition, physical wellness, healthy partnerships, and sleep, stress, and resilience. The Armed Forces Wellness Centers are located at designated military bases in the United States, Europe, Korea, and Japan (see warfighterwellness.org). The Families OverComing Under Stress resiliency training program builds on current strengths and teaches new strategies for military families to respond to and cope with stress and change related to military life and combat injuries through family psychoeducation, communication, goal setting, problem solving, and managing trauma and loss. The program is established at designated United States military bases and has an interactive online platform (see focusproject.org). The Home Base for SMVs and military families is a national program that offers a range of clinical care, wellness, and education programs to military families. The Home Base Resilient Family program is designed to reduce the impact of stress through a variety of mind-body techniques as well as skill-building exercises to improve medical symptoms, mood, wellbeing, communication, peer connections, nutrition, sleep, and physical activity (see homebase.org).

### Sustainability and limitations

Change will occur across the Defense Health Agency requiring DIN leadership to ensure that the FWP survives without losing the essential components or ability to evaluate program outcomes and effectiveness. The FWP was established during a time of budget limitations across the Defense Health Agency. As such, the program was established with minimal clinician or financial burden as a priority to safeguard sustainability. For example, the measures are easily accessed on the TBI Portal, completion is self-paced and brief, the TBI Portal automatically generates symptom severity allowing clinicians to assess severity in live-time, the clinical report is templated, and no additional clinical services or therapies are provided at DIN sites.

In addition, by 01 January 2026, the DIN must be formally established as program of record and fulfill the requirements specified in NDAA FY2025 Sec721. In order to addresses one of three specified duties, the DIN will be required to promote standardization of care across the NICoE, 10 ISCs, and two TBI and Brain Health Clinics as part of the DoD long-term brain health strategy. Annual milestone reviews and briefings to the Committees on Armed Services of the Senate and the House of Representatives will be required covering four elements. One element requires a review and briefing on the number of individuals whose families are able to participate in programs provided by the DIN. These mandated requirements should help facilitate sustainability of the FWP's essential components and uptake of the FWP across the DIN as part of standard TBI care and annual briefing metrics to Congress.

However, the FWP faces certain challenges. At the time of writing this report, there was no additional clinical support for the FWP, delaying the launch of the program at the inaugural sites and efforts to offer individual and dyadic clinical interventions to IPs onsite. In addition, in 2025 the Congressional reporting period for the 15-Year Longitudinal TBI Study concluded and all study activities were closed. In May 2025, the WRNMMC TBICoE site closed due to funding, and the FWP lost research support from the CGFM Study team. The FWP currently has no dedicated research support to establish evaluation or outcomes methodologies, human research protocols, database cleaning and preparation procedures, or outcome statistical analyses.

Data from the FWP across the DIN would be a unique opportunity to explore bidirectional and reciprocal dyadic pathways. For example, exploring how individual, couple, and family level distress and treatment outcomes in military families influence and are influenced by each other. As highlighted earlier, bidirectional and reciprocal family dynamics may have important implications for TBI treatment outcomes and miliary family readiness. Reciprocal modelling statistical methods (e.g., structural equation modeling or two stage least squares) require large sample sizes. The establishment of the FWP across the 13 DIN sites would facilitate access to a large dataset of programmatic data and support reciprocal modelling statistical methods.

Expansion of the FWP clinical and research operations will require dedicated long-term funding. To facilitate access to resources for sustainability and growth of the FWP long-term, DIN leadership plans to integrate the FWP into the DIN Translating Research into Practice (TRIP) Initiative. The TRIP is an organizational framework within the DIN established to foster interdisciplinary collaboration between government, academic, and industry partners with the goal of integrating evidence-based and innovative research into the clinical setting. The TRIP framework involves a 4-step process: [1] identify a clinical need, [2] identify an intervention to address the clinical need, [3] implement and evaluate the intervention; and [4] monitor the intervention and adapt as needed. The FWP has completed Step 1 and 2 as part of the 15-Year Longitudinal TBI Study. Steps 3 and 4 will take place as part of the DIN TRIP initiative.

Another trend is the work being done by the DIN Research Advisorate to create a unified platform for institutional regulatory approval processes to facilitate multisite clinical quality improvement and program evaluation initiatives, human research protocols, and research translation activities across the DIN. A unified assessment and regulatory platform for DIN research, clinical, and education initiatives should advance the coordination and administrative processes for the FWP expansion efforts, quality improvement and human participant research initiatives, and health outcomes evaluation and education for warfighters with TBI and their families into the future.

## Conclusion

The negative influence of family distress on warfighter brain health is a factor that is under-appreciated, modifiable, and has the potential for significant impact on warfighter recovery and readiness following a TBI. Warfighters with TBI and comorbidities have stated a preference for couples and family therapy over individual treatment due to its positive impact on family relationships and quality of life outcomes (91). However, family members of injured warfighters are often invisible partners in the warfighter's recovery and return to duty (81, 92). Their care provision efforts often go unnoticed and their physical, psychological, social, and care provision needs often go unmet. In recent research, one family caregiver noted that “Far too often, the veteran is the only one who is made to be the primary person in need of support. If you’re not talking about the other people in their life, you are missing a giant piece of the puzzle” (p. 16) (37). Quoting Dr. Jill Biden (First Lady 2021-2025) from a virtual Joining Forces event (07 April 2021) “We have an all-volunteer force—and it continues only because generations of Americans see the honor, dignity, and patriotism of military service. How can we hope to keep our military strong if we don’t give our families, survivors, and caregivers what they need to thrive?”

In 2021 the United States DoD released the Warfighter Brain Health Initiative Strategy and Action Plan. In that plan, the need to further understand the late effects that prevent warfighters from returning to optimal brain health following TBI was identified as a line of effort. Findings from individual and dyadic analyses as part of the Congressionally driven 15-Year Longitudinal Study (NDAA FY2007 Sec721 Public Law 109-364) and broader literature add to that initiative by highlighting that a warfighter's ability to recover from a TBI is likely to be compromised when there are high levels of distress and dysfunction in their home environment.

In addition, collaboration across the Military Health System Centers of Excellence has been emphasized as a priority for research and clinical applications. The translational research discussed in this report was the result of a successful, productive, and collegial interdisciplinary collaboration between two Centers of Excellence aimed at advancing warfighter brain health research, clinical care, and education; i.e., the TBICoE and NICoE/DIN. The result of this collaborative effort was an empirically driven and innovative care initiative for DoD beneficiaries that has the potential for a direct effect on the health, wellbeing, and readiness of warfighters and their family members. This successful research to clinical application is continuing with new Military Health System collaborations being established between DIN treatment centers and health care services on military bases and in the community. Continued operations and expansion of the FWP across the DIN will open the door for family wellness to have a long-term place in DoD TBI treatment programs as a holistic, family-centered interdisciplinary model of care for warfighter brain health and return to duty following a TBI, and healthy, resilient, and military ready families.

## Author's note

The views expressed in this report are those of the authors and do not necessarily represent the official policy or position of the Defense Health Agency, Department of Defense, or any other U.S. government agency. This work was prepared under Contract HT0014-22-C-0016 with DHA Contracting Office (CO-NCR) HT0014. For more information, please contact dha.TBICOEinfo@health.mil.

## Footnotes

^1^Brickell TA, Wright MM, Sullivan JK, Ivins BJ, Varbedian NV, Byrd AM, et al. Longitudinal pediatric symptom trajectories and parental risk factors for psychological distress in children of warfighters with traumatic brain injury. *Military Psychology*. (2025).

^2^Brickell TA, French LM, Lippa SM, Wright MM, Baschenis SM, Sullivan JK, et al. Unhealthy family functioning is strongly associated with warfighter brain health following traumatic brain injury in United States service members and veterans. *Frontiers in Neurology*. (2025).
